# Reliability and validity of the Chinese version of the Family Resilience Inventory

**DOI:** 10.3389/fpsyg.2025.1456132

**Published:** 2025-05-22

**Authors:** Xiaobing Xu, Yan Wang, Juntong Meng, Wanlu Cao, Ye Liu

**Affiliations:** ^1^School of Nursing, Qingdao University, Qingdao, China; ^2^Qilu Hospital, Shandong University (Qingdao), Qingdao, China; ^3^School of Nursing and Rehabilitation, Shandong University, Jinan, China

**Keywords:** family resilience, inventory, validity, reliability, chronic disease

## Abstract

**Introduction:**

Family resilience plays a crucial role in helping patients with chronic diseases manage their conditions and maintain overall well-being. The Family Resilience Inventory (FRI) assesses resilience across generations with a focus on protective and promotive factors. However, the FRI has not been translated into Chinese or validated for use among families managing chronic diseases. Therefore, this study aims to test assess the reliability and validity of the Chinese Version of the Family Resilience Inventory (FRI-C) among patients with chronic diseases.

**Methods:**

The Chinese version of the FRI was obtained through standardized forward translation and cultural adaptation. We recruited 307 patients with chronic diseases from a tertiary hospital in Qingdao, Shandong Province, to complete the FRI. Reliability was assessed using calculating Cronbach’s alpha and Guttman split-half reliability. Construct validity was evaluated through the correlation of the FRI-C with the shortened Chinese version of the Family Resilience Assessment Scale (FRAS-C). Confirmatory factor analysis (CFA) was used to validate the structural and discriminant validity of the questionnaire.

**Results:**

The FRI-C had a Cronbach’s alpha coefficient of 0.964 with 0.959 and 0.952 for the two factors. The split-half reliability was 0.716 for the total scale and 0.961 and 0.943 for the two factors. The FRI-C scales and factor scores were significantly correlated with the FRAS-C total score (r values between 0.692 and 0.810, *p* < 0.01). CFA revealed that *χ*^2^/df, goodness-of-fit index, incremental fit index, normed fit index, Tucker–Lewis index, comparative fit index, and root-mean-square error of approximation were all within the acceptable range.

**Conclusion:**

The FRI-C demonstrated strong reliability and validity among patients with chronic diseases and it can be used to evaluate family resilience.

## 1 Introduction

Family resilience is the collective ability and process by which a family navigates stressful events encountered, overcomes adversity, and restores healthy family adaptability (Walsh, 2016a, 2016b). It is crucial for family and individual adaptation during severe crises (Figley and McCubbin, 2016). Family resilience is often considered to be a key determinant of family adaptability in patients with chronic diseases, positively influencing illness management, emotional well-being, and caregiving burden reduction (Zhang et al., 2022, 2023).

Chronic diseases mainly include cardiovascular diseases, chronic respiratory diseases, tumors, and diabetes (Hunter and Reddy, 2013). Advancing medical care and an aging population are driving the rising prevalence of chronic diseases in China. The survey shows that the deaths of patients with chronic diseases accounted for 87% of the total number of deaths in China, and the disease burden accounted for approximately 70% of the total disease burden in China (Yuan et al., 2024). Chronic diseases have a complex etiology and long cycles and often co-occur with multiple diseases, imposing a significant burden on individuals and families. Family serves as the main social environment for patients, with its function and adaptability influencing their well-being and that of other members (Goldberg-Looney et al., 2017). High-resilient families help patients manage chronic diseases effectively and support the healthy development of the family (Zhang et al., 2022, 2024).

However, accurate and effective measurement tools are essential for family resilience assessment and intervention. Various tools are available for this purpose. The Chinese version of the Walsh Family Resilience Questionnaire (Wang and Lu, 2023), its revised version (Li and Li, 2021) and the Chinese shorted version of the Family Resilience Assessment Scale (FRAS-C) (Li et al., 2016) are commonly used in China. The first two contain 26 items, and while 32 items are included in the latter to assess the current state of family resilience. However, none consider the influence of family traditional culture. These versions include numerous items, potentially increasing the response burden for participants. Furthermore, while extensive research examines the intergenerational transmission of negative family patterns (Felitti et al., 2019), less attention has been given to how promoting and protective factors compare between families of origin and their current families. The emergence of the Family Resilience Inventory (FRI) addresses existing gaps. The emergence of the Family Resilience Inventory (FRI) filled the gaps. The FRI was developed by Burnette et al. (2020), which measures resilience in both the current family and family of origin, with a strong emphasis on protective and promoting factors. The FRI focuses on both the presence and absence of protective and promotive factors in families of origin and current families, as well as their transmission across generations. For example, families that prioritize traditional culture actively pass it on to the next generation. However, research on the reuse of the FRI remains unknown. In addition, the family-centered concept in China, emphasis on ethical norms, defined role norms for family members, parent–child relationship as the core, and cultural factors such as filial piety foster resilience and help troubled families overcome difficulties (Yao, 2017). However, the FRI is unavailable in Chinese and has not been used in families managing chronic diseases. Further research is needed to better understand the applicability and effectiveness of the FRI for non-Western cultures.

Therefore, this study aims to translate the FRI into Mandarin Chinese and validate its use among Chinese patients with chronic diseases. This study could provide a brief assessment tool for the measurement of family resilience in Chinese patients with chronic diseases, laying a foundation for future investigations and intervention studies.

## 2 Materials and methods

### 2.1 Participants

Patients with chronic diseases who visited a tertiary hospital in Qingdao City, Shandong Province, were selected using the convenience sampling method from October 2023 to January 2024 for the survey.

The inclusion criteria for patients with chronic disease included: (1) diagnosis of at least one chronic disease (According to the classification criteria in the Chinese version of the Eleventh Revision of the International Classification of Diseases (ICD-11), the scope of chronic diseases includes 20 conditions, including hypertension, diabetes, coronary heart disease, chronic obstructive pulmonary disease, among others.); (2) Understood the purpose of the study and volunteered to participate in the study. The exclusion criteria included the following: (1) mental impairments and (2) poor communication or inability to communicate properly.

According to Wu (2010), CFA requires a minimum sample size of 200 cases; therefore, the final sample size should be ≥200 cases. Therefore, to account for invalid questionnaires (such as short completion or incomplete answers, and so on), 320 questionnaires were distributed. Valid questionnaires (*n* = 307) were completed, yielding a response rate of 95.9%.

### 2.2 Study tools

#### 2.2.1 Family Resilience Inventory

The FRI was developed by Burnette et al. (2020) in 2019 based on previous qualitative research. It filled the gap where culture-based and experience-based measures of family resilience are missing. The FRI comprised two subscales measuring distinct dimensions of family resilience—resilience in the current family and resilience in the family of origin. Each subscale has 20 items and could be used in combination or separately. The FRI scores are dichotomous, with “yes” = 1 and “no” = 0. The total score for each subscale ranges from 0 to 20, where higher scores indicate greater family resilience. The Cronbach’s *α* coefficient for the original scale was 0.92.FRI was not only a tool to measure family resilience, but also to document the facilitators of the family and the absence of these factors, with the aim of assessing the family resilience of the subject’s current family and family of origin, so that protective factors in the family can be assessed across generations.

#### 2.2.2 The shortened Chinese version of the family resilience assessment scale (FARS-C)

The FARS-C is a 32 item self-reported scale that assesses family resilience (Li et al., 2016). It is divided into three dimensions, namely family communication and problem-solving (FCPS), utilizing social resources (USR), and maintaining a positive outlook (MPO). Items are rated on a 4-point scale from strongly disagree (1) to strongly agree (4). The total score can range from 32 to 128, with higher scores indicating greater family resilience. The total score of each subscale is the sum of the scores of its items. Higher scores on the FCPS subscale represent better interaction and communication skills of their families. The higher the FCPS subscale score, the better the family interaction and communication ability. A higher score on the MPO subscale represent the family’s optimistic attitude toward coping with adversity. The scale has evidence of good reliability and validity in the Chinese population (Jiang et al., 2023; Li et al., 2024). In this study, the shortened FARS-C was chosen as a validation instrument.

#### 2.2.3 Demographic variables

The demographic variables obtained from the participants included age, sex, marital status, ethnicity, educational level, professional status, medical insurance, per capita monthly income, living conditions, and chronic disease status.

### 2.3 Translation and cross-cultural adaptation questionnaires

Permission to translate the FRI into Chinese was obtained from the original author, Burnette. The translation and revision of the FRI followed the Brislin translation model (Brislin, 1976). First, a researcher and a nursing graduate student, both proficient in English and having passed the CET-6, separately translated the FRI into Chinese versions I and II, respectively. Another researcher then merged these translations to create the Chinese version III. A bilingual and bicultural nursing specialist and a doctor of nursing practice independently back-translated the FTI into English, producing back-translated versions I and II. The research team discussed and integrated these back-translations to form back-translation III. The back-translated version III was sent to the original authors for review. Questionable areas were discussed and revised until semantic consistency with the original instrument was achieved, resulting in the first draft of the Chinese version of the FRI (FRI-C).

To culturally adapt the Chinese version of the FRI, we invited nine nursing experts to review the first draft. Afterward, we revised the items with translation errors or semantic ambiguities, such as combining entries 19 and 20 in the subscale into one entry based on their recommendations and specialist opinions. Thus, the FRI-C was created, comprising 38 items across two subscales, each containing 19 entries.

### 2.4 Data analysis

The demographic data were analyzed using the descriptive statistics of means and standard deviation (SD), frequencies and percentages.

Item analysis was performed using the CR (critical ration) method, which ranked the family resilience of all study participants from high to low, with the top 27% classified as the high group and the bottom 27% as the low group. The level of significance of the difference between the two was calculated using two independent sample t-tests. The resulting *t*-value is the CR value. Items were retained if the CR value > 3 and the difference was statistically significant (*p* < 0.05) (Wu, 2010).

Since FRI was developed to measure two aspects of resilience established through prior qualitative research, CFA was performed to validate its two-factor structure. To assess the construct validity of the FRI for Chinese culture, the 38 FRI items were subjected to CFA using Amos 26.0. The maximum likelihood method was used for model fitting. The evaluation indicators and acceptable criteria of model fitting mainly include chi-square/freedom degree (*χ*^2^/df) < 3.00, goodness-of-fit index (GFI) > 0.80, incremental fit index (IFI) > 0.80, normed fit index (NFI) > 0.80, Tucker–Lewis index (TLI) > 0.80, comparative fit index (CFI) > 0.80, and root-mean-square error of approximation (RMSEA) < 0.08 (Doll et al., 1994).

The combined reliability, average variance withdrawal (AVE), and the square root of the AVE were calculated according to the path coefficients of the model in the CFA to evaluate the convergent and discriminative validity of the scale. The combined reliability needs to be >0.7, AVE > 0.5, and the square root of the AVE exceeds the correlation coefficient between related factors (Fornell and Larcker, 1981).

The concurrent criterion validity was examined through the correlation of the FRI total scale and the subscales with the shortened FARS-C using Pearson’s correlation coefficients to evaluate these univariate relationships. The coefficients were small (0.1 < |r| < 0.3), medium (0.3 < |r| < 0.5), and large (|r| > 0.5). Cronbach’s alpha and the Guttman split-half reliability coefficient were used to analyze the internal consistency and split-half reliability, respectively.

## 3 Results

### 3.1 General information

Table 1 shows the characteristics of patients with chronic diseases. The age of patients with chronic diseases ranged from 20 to 85 years (mean = 46.04 ± 14.73), with the majority distributed in the 41 ~ 50 (32.2%) age group. Among the participants, 34.2% had a single chronic disease, while 65.8% had multiple chronic diseases.

**Table 1:** Sociodemographic and family characteristics of participants.

Characteristics	Total (*N* = 307) X ± S/frequency (%)
Age (year)	46.04 ± 14.73
Age groups	
≤30	62(20.2)
31 ~ 40	37(12.1)
41 ~ 50	99(32.2)
51 ~ 60	72(23.5)
≥61	37 (12.1)
Gender	
Male	135 (44.0)
Female	172 (56.0)
Marital status	
Unmarried/divorced	56 (18.2)
Married	249 (81.1)
Widowed	2 (0.7)
Ethnic	
Han Chinese	306 (99.7)
Other	1 (0.3)
Religious	
Yes	28 (9.1)
No	279 (90.9)
Educational level	
Elementary school and below	25 (8.1)
Junior high school	88 (28.7)
Senior high school	55 (17.9)
College and above	139 (45.3)
Professional status	
Incumbency	147 (47.9)
Sick leave	1 (0.3)
Retirement	64 (20.8)
Unemployed or other	95 (30.9)
Medical insurance	
Yes	279 (90.9)
No	28 (9.1)
Per capita monthly income	
<2000	41 (13.4)
2000–3,999	108 (35.2)
≥4,000	158 (51.5)
Living condition	
Living alone	53 (17.3)
Living with a spouse	138 (45.0)
With children and spouses and parents	94 (30.6)
Other	22 (7.2)
Chronic disease	
1	105 (34.2)
2 or more	202 (65.8)

### 3.2 Validity

#### 3.2.1 Item analysis and differentiation

The mean score of each item was 0.39–0.65, and a standard deviation of 0.48–0.50. The item-total score Pearson correlation analysis revealed that each item was positively correlated with the total score, with correlation coefficients ranging from 0.540 to 0.771, and they were statistically significant (*P* < 0.01). The independent sample *t*-test results comparing the high (top 27%) and low groups (bottom 27%) showed CR values for each item ranging from-10.726 ~ −40.187 (*P* < 0.01) (see Table 2).

**Table 2:** Comparison of scores of each item of the Chinese version of the Family Resilience Inventory between high group and low group.

Item	Item content	CR1	CR2
1	My family and I know what’s expected of us.	−40.187^**^	−19.716^**^
2	We all value education.	−17.985^**^	−21.737^**^
3	We express love and affection freely. (e.g., hugging, kissing, saying “I love you”)	−13.894^**^	−23.763^**^
4	My family is full of laughter.	−16.725^**^	−15.427^**^
5	We have a lot of family time together. (e.g., doing activities, eating, spending quality time)	−20.735^**^	−13.958^**^
6	Adult arguing is kept away from children.	−24.070^**^	−21.573^**^
7	I feel my family is stable, safe, and predictable.	−13.612^**^	−15.704^**^
8	We have family members to look up to (role models).	−16.833^**^	−20.416^**^
9	We support each other’s family activities and goals.	−14.496^**^	−19.716^**^
10	We do not tolerate violence against any member of the family.	−15.986^**^	−13.206^**^
11	We work together to help each other and to complete goals.	−19.635^**^	−10.726^**^
12	We have strong values that guides our actions.	−17.985^**^	−16.725^**^
13	We respect all family members. (including elders, women, men, and children)	−21.609^**^	−17.361^**^
14	We are close knit.	−17.598^**^	−14.596^**^
15	We get together a lot for birthdays, holidays, meals, and special events.	−19.835^**^	−14.596^**^
16	We are stronger in tough times.	−16.132^**^	−17.464^**^
17	We come together during hard times, rather than going our separate ways.	−18.052^**^	−13.526^**^
18	We prioritize the needs of children over the needs of adults.	−17.985^**^	−14.041^**^
19	We pass on the traditional culture.	−17.985^**^	−17.361^**^

^**^Indicates that *P* < 0.01. CR1 and CR2 represent the scores of high and low groups on each item in the two dimensions, respectively.

#### 3.2.2 Structural validity

**Figure 1:**
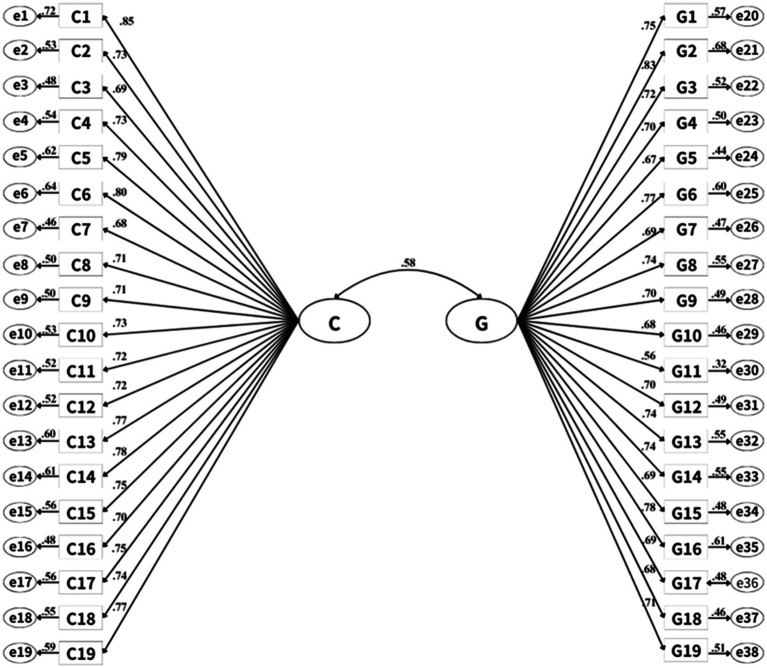
Standardized two-factor structural equation model of Family Resilience Inventory-Chinese version (loadings > 0.55 are significant for *p* < 0.01). C is the dimension of “In my current family unit,” G is the dimension of “In my family growing up”. C1 ~ C19 and G1 ~ G19 represent specific items.

Since the original scale was divided into two dimensions, CFA was conducted using the AMOS software data from 307 questionnaires to further verify its structural validity. The model fit indices were as follows: *χ*^2^ = 928.852, *χ*^2^/df = 1.399 < 3, *P* < 0.001, GFI = 0.862, IFI = 0.964, NFI = 0.885, TLI = 0.962, CFI = 0.964, RMSEA = 0.036. These indices indicated an acceptable model fit. The standardized regression coefficients ranged from 0.56 to 0.85 (Figure 1).

#### 3.2.3 Convergent and discriminative validity

The results showed that combined reliability values were 0.952 and 0.959, both exceeding 0.7, while the AVE values were 0.551 and 0.553, both above 0.5. The square roots of the AVE values were 0.744 and 0.715, respectively, surpassing the correlation coefficients of the relevant components. These findings indicate the strong convergent and discriminative validity of the FRI-C.

#### 3.2.4 Concurrent criterion validity

Using the FRAS-C as the efficacy criteria, the overall sample was tested for the correlation validity of the criteria. Pearson correlation analyses revealed significant correlations between the total score of the scale, its factors, the FRAS-C, and its subscales, with correlation coefficients ranging between 0.682 and 0.810 (*P* < 0.01). Table 3 shows the results.

**Table 3:** Correlation between each factor of the Family Resilience Inventory and the calibration variables.

Calibration variables	FCPS	USR	MPO	FRAS-C	In my current family unit	In my family growing up	Total score
FCPS	1	0.957^**^	0.975^**^	0.998^**^	0.732^**^	0.689^**^	0.805^**^
USR		1	0.965^**^	0.969^**^	0.739^**^	0.684^**^	0.807^**^
MPO			1	0.986^**^	0.728^**^	0.682^**^	0.799^**^
FRAS-C				1	0.737^**^	0.692^**^	0.810^**^
In my current family unit					1	0.558^**^	0.888^**^
In my family growing up						1	0.878^**^
Total score							1

^**^Indicates that *P* < 0.01.

FRAS-C is the shortened Chinese version of the Family Resilience Assessment Scale; FCPS is the Family Communication and Problem Solving subscale; USR is the Utilizing Social Resources subscale; MPO is the Making a Positive Outlook subscale.

### 3.3 Reliability

#### 3.3.1 Internal consistency confidence

Cronbach’s *α* coefficient for the total FRI scale was 0.964, while the two subscales had coefficients of 0.959 and 0.952, respectively.

#### 3.3.2 Split-half reliability

The split-halt reliability of the FRI total scale was 0.716, while both subscales had values of 0.961 and 0.943, respectively.

## 4 Discussion

### 4.1 The significance of the Chinese version of FRI

Chronic diseases are the leading cause of death globally, accounting for 74% of all fatalities (World Health Organization, 2022). The diagnosis and treatment of chronic diseases not only affect the physical and mental health of patients but also expose their families to several challenges, such as the financial burden on the family (Darwish et al., 2020), the negative emotions of the main caregiver, and reduced quality of life (Intas et al., 2020). Research reveals that family resilience is crucial for both family and individual adaptation during severe crises (Figley and McCubbin, 2016). Accurate and effective measurement tools are essential for family resilience assessment and intervention. Some methods can be used to assess family resilience in chronic disease contexts. However, variations in research backgrounds, subjects, and tools lead to inconsistent results, limiting understanding of the promoting factors of family resilience and the intergenerational relationship. Therefore, this study translated the FRI compiled by Burnette et al. (2020) into Chinese, applied it to Chinese patients with chronic diseases, and validated its reliability and validity.

The FRI-C is designed to assess family resilience in both the current family (19 items) and family of origin (19 items). These subscales can be used individually or together to identify intergenerational patterns. It is the shortest Chinese family resilience scale with strong clinical applicability. The difference is that the FRI includes the protective and promotive factors of family across these generations. Participants only need to rate the items “yes/no” (Burnette et al., 2020), enabling quick identification of missing protective factors and facilitating targeted interventions. Family resilience is a dynamic process, given the ongoing challenges associated with chronic diseases (Faccio et al., 2018). The FRI introduces an innovative approach to studying intergenerational family resilience. This provides an efficient and accurate tool for assessing family resilience in Chinese patients with chronic diseases. It provides direction and reference for the prevention and intervention of family crises in chronic disease care.

### 4.2 The psychometric properties of the Chinese version of the FRI

In the items analysis, the Pearson correlation coefficients of the item-total score of FRI-C ranged from 0.540 to 0.771, all exceeding 0.4 (Yang et al., 2019), indicating a strong internal aggregation degree of the scale. The CR values for all 38 items were >3 (*P* < 0.05), demonstrating good item differentiation.

Reliability means the stability, equivalence, and internal consistency of the results measured using a measurement tool (Hao and Jingwu, 2006). Generally, Cronbach’s *α* coefficients above 0.7 are considered acceptable, while subscale values should ideally exceed 0.6. The reliability coefficients of FRI-C fall within the acceptable range, indicating its strong reliability. The consistency reliability results of this study were slightly higher than those of the original FRI. This may be due to different cultural backgrounds and study populations.

Validity refers to the validity and accuracy of the measurement results. CFA revealed a well-fitted model, indicating that FRI-C has good construct validity (Lobefaro et al., 2022). The FRI-C included 38 items and two subscales (19 entries each), measuring distinct dimensions of family resilience—resilience in the current family and resilience in the family of origin. It is consistent with the structure of the original scale. The combined reliability values for the two-factor model were 0.952 and 0.959, with AVE values of 0.551 and 0.553. The square roots of the AVE values were 0.744 and 0.715, exceeding the correlation coefficients of the correlated components, indicating strong convergent and discriminative validity. Finally, FRAS-C served as the efficacy criteria in this study. Its correlation with the FRI-C total scale and two subscales ranged from 0.692 to 0.810, indicating the suitable concurrent validity of FRI-C. The high reliability and validity of FRI-C in chronic stress situations suggest its universality across disease scenarios. This is consistent with the resilience framework proposed by Kumpfer (2002), in which adversity may stimulate their underlying resilience.

## 5 Limitations

Although this study contributes significantly to the literature, it has some limitations. First, the Chinese version of the FRI has only been validated in disease-oriented clinical populations (patients with chronic diseases), limiting its applicability to non-clinical populations facing non-medical adversities (e.g., financial crises, natural disasters) and families affected by other types of illnesses (e.g., acute or psychiatric conditions). Future studies will include general families experiencing normative stressors (e.g., parenting challenges, work–family conflicts), non-clinical families coping with systemic adversities (e.g., unemployment, socioeconomic disparities), and clinical populations with diverse medical conditions (e.g., cancer, mental health disorders) to comprehensively test the broad applicability of FRI-C. Second, the study was conducted on a sample of patients with chronic diseases from a tertiary hospital in Shandong Province, limiting its generalizability. Future studies should adopt multi-center designs encompassing diverse geographical regions and socioeconomic backgrounds. Finally, only CFA was performed in this study without Exploratory Factor Analysis. Since both of them require different samples (Wang et al., 2024), subsequent studies should use two independent samples to conduct both analyses simultaneously for validation.

## 6 Conclusion

The results of this study show that the FRI-C has strong reliability and validity in the Chinese population with chronic diseases, including two factors: “In my current family unit” and “In my family growing up.” The FRI-C serves as a stable, reliable, concise, and validated tool for measuring family resilience in individuals with chronic diseases. It captures family protective and promotive factors, as well as their absence. Further consideration should be given to applying this tool in longitudinal and intervention studies.

## Data Availability

The datasets generated during this study are not publicly available to protect the privacy of participants. All individuals contributed data under voluntary confidentiality agreements that prohibit open sharing. Requests to access the datasets should be directed to Xiaobing Xu: 1097065933@qq.com.
